# Hepatic encephalopathy: A neurochemical, neuroanatomical, and neuropsychological study

**DOI:** 10.1120/jacmp.v7i1.2151

**Published:** 2006-02-21

**Authors:** Nader Binesh, Amir Huda, M. Albert Thomas, Nathaniel Wyckoff, Mary Bugbee, Steven Han, Natalie Rasgon, Pablo Davanzo, James Sayre, Barry Guze, Paul Martin, Fawzy Fawzy

**Affiliations:** ^1^ Departments of Radiological Sciences University of California Los Angeles California 90095; ^2^ Departments of Hepatology University of California Los Angeles California 90095; ^3^ Departments of Psychiatry University of California Los Angeles California 90095; ^4^ Department of Physics California State University Fresno California 93740 U.S.A.

**Keywords:** cerebral metabolites, PRESS, glutamine/glutamate, myo‐inositol, choline, hyperintensity, neuropsychology

## Abstract

Hepatic encephalopathy (HE) is normally diagnosed by neuropsychological (NP) tests, which are not very specific and do not reveal the underlying pathology. Magnetic resonance imaging (MRI) and spectroscopy (MRS) of the brain offer alternative and possibly more specific markers for HE. These methods were applied in conjunction with NP testing in order to determine their usefulness in the identification of HE and to understand the pathogenesis of HE more clearly. MR imaging and spectroscopy examinations, in addition to a battery of 15 NP tests, were administered to investigate 31 patients awaiting liver transplantation and 23 healthy controls. MR image intensities from the globus pallidus region were calculated and normalized to those of the thalamus. Absolute concentrations and ratios with respect to creatine (Cr) of several metabolites were computed from MR spectra. The MR data were correlated with the results of NP tests. The patients showed impairment in NP tests of attention and visuospatial and verbal fluency. In $T_{1}$ weighted MRI, the relative intensity of the globus pallidus with respect to that of the thalamus region was significantly elevated in patients and correlated (negatively) with three NP tests (Hooper, FAS, and Trails B). The absolute concentrations of myo‐inositol (mI) and choline (Ch) were significantly reduced in three brain regions. In addition, the absolute concentrations of glutamine (Gln) and combined glutamate and glutamine (Glx) were increased in all three locations, with Gln increase being significant in all areas while that of Glx only in the occipital white matter. In summary, this study partially confirms a hypothesized mechanism of HE pathogenesis, an increased synthesis of glutamine by brain glutamate in astrocytes due to excessive blood ammonia, followed by a compensatory loss of myo‐inositol to maintain astrocyte volume homeostasis. It also indicates that the hyperintensity observed in globus pallidus could be used as complementary to the NP test scores in evaluating the mental health of HE patients.

PACS number: 87.61.Pk

## I. INTRODUCTION

Hepatic encephalopathy (HE) is a syndrome of potentially reversible neurological deficits commonly found in patients with chronic liver disease. There are two recognized classes of this illness: overt and minimal HE. Overt HE is characterized by a generalized movement disorder and the alteration of consciousness. Its symptoms range in severity from disturbed sleep, irritability, forgetfulness, and tremor in the syndrome's mildest manifestation (grade 1) to coma and severe neurological dysfunction in the syndrome's most severe form (grade 4).^(^ ^1^ ^,^ ^2^ ^)^ Minimal HE (also known as subclinical HE) is a subtle encephalopathy. Patients with minimal HE suffer deficiencies in neurocognitive, psychomotor, and visuospatial functions (as measured by specific neuropsychological (NP) test abnormalities), while appearing normal on standard NP examinations.^(^ ^3^ ^–^ ^5^ ^)^ Although NP tests constitute the current diagnostic standard for minimal HE,^(^ ^3^ ^,^ ^6^ ^–^ ^9^ ^)^ the results of these tests are not very specific and do not reveal the underlying neurochemical pathophysiology. Magnetic resonance imaging (MRI) and spectroscopy (MRS) examinations of the brain have revealed some characteristic markers of HE; these methods may complement the NP examination.

The brains of HE patients often display a bilateral and symmetric hyperintensity of the globus pallidus on $T_{1}$‐weighted MR scans. However, on $T_{2}$‐weighted images, the globus pallidus appears normal, suggesting that the hyperintensities result from the deposition of a paramagnetic material in this region of the brain.^(10)^ Abnormal appearance of the basal ganglia has been related to severe liver failure.^(^ ^11^ ^,^ ^12^ ^)^ An MRI study of 26 patients with biopsy‐proven cirrhosis found a greater contrast between the signal intensities of the globus pallidus and surrounding white matter in patients with neuropsychiatric impairment than in unimpaired patients.^(13)^ Atomic absorption spectrometry of samples of globus pallidus obtained from autopsy of patients with chronic liver disease revealed elevated levels of manganese and copper, with manganese being the dominant one.^(14)^ Hauser et al. showed correlation between blood manganese levels and hyperintensity of globus pallidus,^(15)^ suggesting the manganese as the neurotoxin that causes the globus pallidus hyperintensity in chronic liver disease.^(16)^

MRS changes in various brain locations have been associated with HE.^(^ ^17^ ^,^ ^18^ ^)^ Recently, MRS has found patients with either overt or minimal HE to have altered levels of glutamine/glutamate (Glx), myo‐inositol (mI), and choline (Cho) compounds.^(^ ^9^ ^,^ ^19^ ^–^ ^25^ ^)^ Increased intracellular concentration of Glx in HE is due to the up‐regulation of glutamine (Gln) synthetase that occurs in the presence of increased ammonia concentrations.(26) Hence, the increase of Glx reflects a disturbance of cell volume homeostasis. Elevated Glx levels also correlate with the hyperintense lesions in the basal ganglia documented by MRI.^(25)^ Amino acids and mI are polyols^(9)^ (organic osmolytes) that accumulate intracellularly to regulate cell volume. The intracellular increase of Gln is thought to lead to a decline of mI through a compensatory mechanism.^(26)^ Cho includes phosphoryl choline and glycerylphosphoryl choline. Although the pathophysiological importance of Cho is not completely understood,^(^ ^27^ ^–^ ^30^ ^)^ we suspect that it functions as a cerebral osmolyte,^(9)^ in light of the liver's role in the biosynthesis of glycerophosphate and lipids.

The goals of the present study were the following: (1) to quantify the cerebral proton metabolites in patients awaiting liver transplantation, with minimal or grade 1 HE, and healthy controls; (2) to explore regional differences between the metabolite levels in the anterior cingulate, occipital white matter, and basal ganglia of patients and compare them to healthy controls; (3) to investigate the relative increase in MRI signal intensity of the globus pallidus in patients as compared to healthy controls; and (4) to establish whether there are correlations between MRI findings, MRS findings, and NP tests of HE patients.

## II. METHODS

### A. Human subjects

The study population was comprised of 31 (18 male and 13 female) patients and 23 (13 male and 10 female) healthy controls. The range of patients’ ages was 35 to 71 years, with the average of 51, while that of healthy controls was 34 to 78, with the average of 54. Because our earlier investigations had suggested that the minimal HE grading could not reliably exclude mild HE,^(^ ^20^ ^,^ ^21^ ^)^ the current study focuses on eligible patients with hepatitis C or alcoholic cirrhosis awaiting organ liver transplantation, even without histories of overt HE. The inclusion criteria included being listed for liver transplantation and having a United Network Organ Sharing status of 3 or greater; the exclusion criteria were lack of fluency in the English language (affecting NP tests) and claustrophobia during the MR examination. The diagnoses of the patients were as follows: 24 hepatitis C (7 used drugs), 14 alcoholic cirrhosis, 2 hepatitis B, and one acute liver failure.

### B. MRI/MRS acquisition and processing

**Figure 1:**
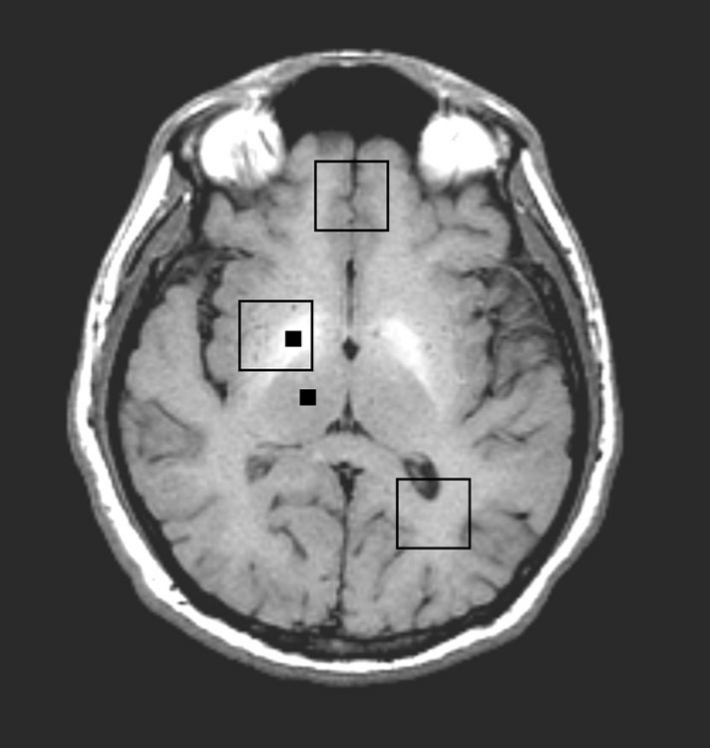
$T_{1}$‐weighted MRI of a 47‐year‐old patient. The two small black squares are the locations used for hyperintensity calculations. The three large squares show the location of the MRS voxels.

All the scans were performed on a 1.5T GE MRI scanner (GE Medical Systems, Waukesha, WI) with “echo‐speed” gradients, using a head transmit/receive coil. The imaging protocol was comprised of a 3D localizer, $T_{1}$‐weighted axial images (TR/TE=800/8ms, 35 slices, 4 mm thickness with no gap), and a coronal FLAIR sequence. Those were followed by three series of single voxel PRESS spectroscopy, with the following parameters: TR=3s,TE=30ms,number of averages=64, and voxel size of $2\times 2\times 2\;{\text{cm}}^{3}$. The voxel localization was guided by the $T^{1}$‐weighted axial images as shown in Fig. 1. Note that the figure is schematic, and in actual scan, the center slices for the three voxels differed.

Images were transferred to a Sun Ultra 10 workstation (Sun Microsystems, Palo Alto, CA) and analyzed using an in‐house developed program that makes use of software toolkits developed previously for medical image analysis.^(31)^ These toolkits enable the display and manipulation of medical images, the representation of the anatomic region of interest, and the calculation of the properties of the region of interest. To measure the degree of hyperintensity in patients, we had to calculate the contrast ratios comparing the MRI signal intensity of the globus pallidus to the signal intensities of reference structures. From each subject's axial $T_{1}$‐weighted images, an image clearly displaying the globus pallidus was selected where the selected slice of the globus pallidus was located within the subject's MRS voxel. The right lobe was selected because the basal ganglia spectra were recorded from each patient's right brain. The thalamus was chosen as the reference structure because it has a very smooth and even signal intensity, which makes the results well reproducible. The mean intensity was calculated for all pixels within a 3×3 pixel box located approximately centrally inside the globus pallidus and thalamus. The differences between mean values for the globus pallidus and that of thalamus were evaluated, and normalized, to obtain the hyperintensity ratio, that is,

(1) $$\text{hyperintensity}\;\text{ratio}=\frac{\text{globus}\;\text{pallidus}\;\text{mean}-\text{thalamus}\;\text{mean}}{\text{thalamus}\;\text{mean}}$$

The spectroscopy data were transferred to an SGI™ $O_{2}$ workstation (Silicon Graphics Inc, San Jose, CA) and processed using the LCModel package.^(32)^ The basis set used for quantification was provided by the vendor for a GE 1.5T scanner and TE=30ms. Absolute concentrations were not corrected for $T_{1}$ and $T_{2}$ saturations.

### C. Neuropsychological tests

The clinical and cognitive evaluations were conducted at the time of initial evaluation and included a psychiatric history and the Mini‐Mental Status Examination (MMSE), on which a score of 24 or less suggested cognitive impairment and high‐grade encephalopathy.^(33)^ Thus, all the subjects in this study had scores above 24 to fulfill the criterion for minimal HE. In addition, a battery of NP tests was administered by a clinical psychologist on the same day as the MRS scan, and took approximately 1.5 hours to complete. The battery consisted of the following: Trail Making Test A and B, three subsets from Wechsler Adult Intelligence Scale III edition (Digit Symbol, Digit Span, and Block Design), Stroop (color, word, and interference), Rey Auditory Verbal Learning Test, Controlled Oral Word Association Test (FAS), Grooved Peg Board (dominant and nondominant hand), Hooper Visual Organization Test, and Wisconsin Card Sorting Test (categories and preservative errors).^(33)^ The raw scores were recorded and compared between patients and healthy controls. The patients and healthy controls were not matched for education and background.

### D. Statistical analysis

The mean and standard deviation (SD) of metabolite concentrations and ratios (with respect to creatine (Cr)) were calculated for patients and healthy subjects. To evaluate the hypothesis that the metabolite concentrations are from the same populations, a two‐tailed *t*‐test was performed. Any metabolite differences with p<0.05 was considered to be statistically significant. The MRI intensity ratios were subjected to the same *t*‐test and criteria for significance. Pearson correlation was performed on the patient data to check for any correlations between the metabolite concentrations, MRI intensity ratios, and the NP test scores. A Bonferroni‐like adjustment^(34)^ was done to ensure that all correlations are still significant with the adjusted alpha level.

## III. RESULTS

**Figure 2:**
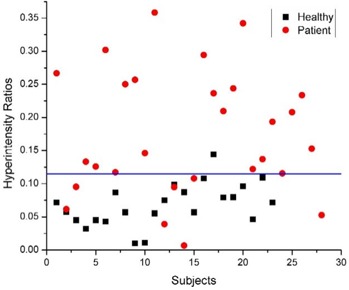
Distribution of hyperintensity ratios in patients (circles) and healthy controls (squares). The horizontal line shows the best approximation for a ratio to separate the two groups.

The hyperintensity of globus pallidus was quite evident from the $T_{1}$‐weighted axial images (Fig. 1). The mean ratio of the pixel intensity of globus pallidus to thalamus in patients was 0.1751(±0.0935), while that in healthy controls was 0.0680(±0.0319). The distribution of the intensity ratios (Fig. 2) is such that no specific ratio (a line drawn parallel to the *x*‐axis) can separate the two groups, although these changes were statistically significant (p<0.0001).

The MRS results are summarized in Tables 1 and 2. A significant decline of choline and myo‐inositol was observed in all three locations of brain examined, in HE patients when compared to healthy controls. In patients, the glutamine concentration had significantly increased at all three locations, but the combined pool of glutamine/glutamate (Glx) displayed a significant increase only in occipital white matter (Table 1. This is possibly due to the fact that the spectra from the occipital white matter had the best quality (shim), which results in low fitting errors.

**Table 1:** Metabolite concentrations

Metabolites	Anterior cingulate gyrus			Right basal ganglia			Left occipital white		
	Mean healthy	Mean patient	*p*‐value	Mean healthy	Mean patient	*p*‐value	Mean healthy	Mean patient	*p*‐value
Ch	1.50	1.23	0.013	1.52	1.28	0.003	1.24	1.04	0.001
Cr	6.46	5.96	0.319	6.89	6.92	0.933	4.44	4.19	0.192
Gln	5.26	8.63	0.004	5.92	8.71	0.001	2.91	5.79	<0.001
Glu	8.22	6.89	0.067	8.35	7.27	0.037	5.26	5.21	0.856
Glx	13.49	14.73	0.376	14.27	15.98	0.087	8.17	11.00	0.001
mI	4.87	2.82	<0.001	3.64	2.30	<0.001	3.77	1.62	<0.001
NAA	6.81	6.08	0.067	6.50	6.08	0.110	6.12	5.60	0.045

**Table 2:** Metabolite ratios with respect to creatine

Metabolite ratios	Anterior cingulate gyrus			Right basal ganglia			Left occipital white		
	Mean healthy	Mean patient	*p*‐value	Mean healthy	Mean patient	*p*‐value	Mean healthy	Mean patient	*p*‐value
Ch/Cr	0.235	0.205	0.001	0.222	0.189	0.002	0.283	0.248	0.003
Gln/Cr	0.882	1.499	0.011	0.858	1.264	<0.001	0.678	1.339	<0.001
Glu/Cr	1.348	1.162	0.144	1.229	1.089	0.09	1.206	1.238	0.635
Glx/Cr	2.230	2.524	0.129	2.087	2.353	0.063	1.883	2.577	<0.001
mI/Cr	0.773	0.461	<0.001	0.529	0.355	<0.001	0.859	0.405	<0.001
NAA/Cr	1.095	1.035	0.249	0.963	0.916	0.377	1.425	1.335	0.265

The NP tests results are tabulated in Table 3. Significant differences were found between the patients and healthy volunteers in the following tests: Trails B, Stroop tests, digit symbol, FAS, and Hooper. These tests were subsequently selected for a correlation (Pearson two‐tailed) study with the MRS and MRI results (Table 4(a), (b), and (c)). Significant (p<0.05) correlations were observed between Hooper and Gln, Glx and mI, only in the occipital white voxel location. The hyperintensity ratio, however, showed significant correlation with three of the NP tests: Trails B, FAS, and Hooper, whose Pearson correlation factors were 0.520,−0.503, and −0.553, respectively. Considering the fact that the higher score on Trails B means lower performance (longer time taken to do the task) as the intensity of the images at basal ganglia increases, the performance on FAS, Hooper, and Trails B declines. The higher intensity ratio also positively correlated with glutamine increase (Pearson correlation factor of 0.571).

**Table 3:** Means and standard deviations (SDs) of the various neuropsychological (NP) tests performed on patients and healthy controls

	Healthy controls		Patients		
NP tests	Mean	SD	Mean	SD	*p*‐value
Trails A	38.48	16.45	43.41	12.48	0.23
Trails B	80.24	25.99	103.04	38.53	0.024
Stroop color	66.13	11.34	77.44	11.51	0.001
Stroop word	47.48	8.75	57.04	11.81	0.002
Stroop interference	121.3	26.06	146.67	27.93	0.002
WCST: Categories	3.16	1.48	3.42	1.66	0.574
WCST: Errors	6.09	3.75	7.81	6.49	0.266
Digit span	16.91	3.29	15.93	2.99	0.272
Digit symbol	55	12.17	43.81	12.18	0.002
RAVLT	45.38	9.71	43.85	10.74	0.611
FAS	37.65	11.81	30.85	9	0.025
GPB dominant	79.48	19.15	84.41	14.22	0.302
GPB nondominant	87.43	21.5	88.92	19.01	0.798
GPB Total	83.46	19.64	86.21	14.71	0.578
Hooper	22.76	3.46	25.65	1.87	0.0005
Block design	34.54	13.96	35.54	9.47	0.772

WCST=Wisconsin Card Sorting Test.

RAVLT=Rey Auditory Verbal Learning Test.

FAS=Controlled Oral Word Association Test.

GPB=Grooved Peg Board Test.

**Table 4:** Pearson correlation between the NP tests showing decline in HE patients and the MR results in three locations: (a) anterior cingulate gyrus, (b) occipital white, and (c) basal ganglia. Only the metabolites that demonstrated significant change in HE patients are shown.

(a)							
Metabolites	Trail B	Stoop color	Stroop word	Stroop interference	Digit symbol	FAS	Hooper
Ch	−0.226	0.255	0.345	0.081	0.036	0.12	−0.059
Gln	0.094	0.15	0.208	0.332	−0.063	−0.274	−0.032
Glu	0.005	0.402	0.303	0.274	−0.041	−0.068	0.263
Glx	0.077	0.327	0.32	0.403	−0.071	−0.251	0.111
mI	−0.349	0.055	0	−0.151	0.183	0.282	−0.046
(b)							
Metabolites	Trail B	Stoop color	Stroop word	Stroop interference	Digit symbol	FAS	Hooper
Ch	0.023	0.216	0.251	0.16	0.013	−0.1	0.35
Gln	0.047	0.145	0.126	−0.083	−0.302	−0.224	−0.652 ^*^
Glu	−0.098	0.11	0.022	−0.146	−0.083	−0.298	−0.229
Glx	0.016	0.156	0.116	−0.111	−0.286	−0.275	−0.631 ^*^
mI	−0.157	−0.143	−0.186	0.053	0.125	0.243	0.543^*^
(c)							
Metabolites	Trail B	Stroop color	Stroop word	Stroop interference	Digit symbol	FAS	Hooper
Ch	−0.246	0.251	0.348	0.116	0.014	0.151	0.050
Gln	0.149	0.116	0.135	0.162	0.006	−0.313	−0.293
Glu	−0.032	0.387	0.305	0.308	−0.067	−0.018	0.357
Glx	−0.015	0.265	0.287	0.420	−0.114	−0.099	0.357
mI	−0.291	0.049	−0.017	−0.195	0.203	0.215	−0.18
MRI Changes	0.520^*^	0.329	0.398	0.195	−0.146	−0.503 ^*^	−0.553 ^*^

^*^ Significant at the 0.05 level.

## IV. DISCUSSION

In this study, globus pallidal image hyperintensity using the $T_{1}$‐weighted MRI images and metabolite changes in three different brain regions of patients with hepatic encephalopathy using MRS were investigated. The cognitive performance was assessed using NP tests, and the test scores were correlated with MRI and MRS analysis. The increase in the image intensity of globus pallidus was quite significant between healthy controls and HE patients (p<0.0001). As some studies have shown, the development of a hyperintensity in the globus pallidus may be secondary to the progression of liver disease and resultant transport of excessive blood ammonia to the brain. Taylor‐Robinson et al. found their MRI‐based globus pallidus contrast measurements to increase with blood ammonia levels and with the severity of liver dysfunction in patients with biopsy‐proven cirrhosis.^(13)^ Kulisevsky and co‐workers found the plasma ammonia level to correlate strongly with the globus pallidus signal in a group of stable cirrhotic patients.^(11)^ To connect this hyperintensity with the severity of HE (or the psychological condition of patients), many researchers have tried to correlate the two,^(35)^ with no definite answers. We used Pearson correlation to investigate the possible connection between the NP tests and hyperintensity in basal ganglia. Interestingly, out of many NP tests, three (FAS, Hooper, and Trail B) showed significant correlation with the hyperintensity ratio. These tests each come from a different domain: Trail B measures attention and speed of processing, FAS measures verbal fluency and language skills, while Hooper is a test of visual organization. Since none of these tests measure any specific task related to basal ganglia, we can only infer that the increase in intensity of globus pallidus correlates with an overall decline in the mental health of HE patients. A larger number of subjects and more rigorous NP testing might be able to identify whether, and to what extent, hyperintensity of the globus pallidus can be used to quantify the severity of HE.

The hyperintensity of the globus pallidus was also correlated with the MRS results from the voxel placed on basal ganglia. This voxel would contain the hyperintense region. The only significant correlation observed was with variation in the glutamine concentration (p=0.009), indicating that the increase in Gln follows the increase in intensity of globus pallidus or vice versa. A possible explanation of this correlation is that both abnormalities are caused by the increase of blood ammonia, which is believed to be the cause of Gln increase^(26)^ as well as deposition of paramagnetic impurities.^(13)^

The current MRS findings, consistent with earlier studies,^(^ ^20^ ^–^ ^25^ ^)^ revealed elevated levels of glutamine and reduced levels of myo‐inositol and choline, in all three regions. Glx showed significant increase in the occipital region, while in the other two locations it showed an increasing trend without statistical significance (Table 1. Glx and Gln recorded in the occipital region showed negative correlation with Hooper, while mI showed a positive one. It appears that a lower result from the Hooper test corresponds to an increase of Gln and Glx and a reduction of mI concentrations. There is a need for further testing to confirm whether Hooper is actually correlating with the increase in Glx or the ammonia toxicity in the brain.

A significant decrease in the N‐acetyl aspartate (NAA) concentration and a nonsignificant decrease in NAA/Cr recorded in the occipital white matter warrant further evaluation. The absolute concentrations that are reported here were not corrected for atrophy, but the ratios are independent of the cerebro‐spinal fluid volumes. The variation in the relative levels of NAA with respect to Cr was not significant in patients when compared to healthy controls in any of the three areas (Table 2.

Reduction of myo‐inositol is consistent with a recent proposed mechanism of HE pathogenesis^(26)^: an intracellular increase of glutamine in astrocytes followed by a loss of myo‐inositol in order to preserve cell volume homeostasis. The increased Gln concentration is thought to arise from an increased synthesis of glutamine from glutamate and excess ammonia following the said hypothesis. We can see that Gln increases and Glu tends to decrease (not significantly) in the brains of HE patients. However, the decrease in choline is not well understood.^(25)^

## V. CONCLUSION

This study combines several individual studies reporting individual facets of HE into one study. It explores regional variations of spectroscopic changes in minimal HE, along with hyperintensity of globus pallidus and correlations of these findings with NP test results. Thus, it reiterates the earlier MRS results of a large decline in mI, a decrease in Cho, and an increase in Glx in the parietal white matter of patients with HE to other areas of the brain as well; the study also partially confirms a hypothesized mechanism of HE pathogenesis, an increased synthesis of glutamine by brain glutamate in astrocytes due to excessive blood ammonia, followed by a compensatory loss of myo‐inositol to maintain astrocyte volume homeostasis. It correlates the hyperintensity observed in the globus pallidus to a general decline in patient performance in a few NP tests. Possible limitations of the present study are the overlap of spectroscopic peaks resulting in large errors in fitting with LC Model, partial volume effects due to the voxel size, and lack of control on matching the education and background of the healthy volunteers with the patients. Considerations of the signal‐to‐noise ratio have prompted the use of 8‐mL voxels. A remedy to this problem is the use of better coils and higher magnetic fields. Higher magnetic fields with better shims can also help in reducing the overlap of the peaks. An alternative method is 2D spectroscopy,^(36)^ using the cross‐peak ratios that are overlap‐free. The next phase of this study using 2D spectroscopy is already underway.

## ACKNOWLEDGMENTS

This work was supported by the NIH grants (MH58284 and MH065695). The authors would like to thank Dr. Mathew Brown for his help with the MRI analysis software and Dr. Michael Green for valuable discussions on the neuropsychological test score analysis.
